# Neuromuscular impairments of cerebral palsy: contributions to gait abnormalities and implications for treatment

**DOI:** 10.3389/fnhum.2024.1445793

**Published:** 2024-09-18

**Authors:** Kylie Clewes, Claire Hammond, Yiwen Dong, Mary Meyer, Evan Lowe, Jessica Rose

**Affiliations:** ^1^Motion and Gait Analysis Lab, Lucile Packard Children’s Hospital, Stanford Medicine Children’s Health, Palo Alto, CA, United States; ^2^Department of Mechanical Engineering, Rice University, Houston, TX, United States; ^3^Department of Civil and Environmental Engineering, Stanford University, Stanford, CA, United States; ^4^Department of Orthopedic Surgery, Stanford University, Stanford, CA, United States

**Keywords:** cerebral palsy, brain injury, neuromuscular impairments, musculoskeletal impairments, gait abnormalities

## Abstract

Identification of neuromuscular impairments in cerebral palsy (CP) is essential to providing effective treatment. However, clinical recognition of neuromuscular impairments in CP and their contribution to gait abnormalities is limited, resulting in suboptimal treatment outcomes. While CP is the most common childhood movement disorder, clinical evaluations often do not accurately identify and delineate the primary neuromuscular and secondary musculoskeletal impairments or their specific impact on mobility. Here we discuss the primary neuromuscular impairments of CP that arise from early brain injury and the progressive secondary musculoskeletal impairments, with a focus on spastic CP, the most common form of CP. Spastic CP is characterized by four primary interrelated neuromuscular impairments: 1. muscle weakness, 2. short muscle-tendon units due to slow muscle growth relative to skeletal growth, 3. muscle spasticity characterized by increased sensitivity to stretch, and 4. impaired selective motor control including flexor and extensor muscle synergies. Specific gait events are affected by the four primary neuromuscular impairments of spastic CP and their delineation can improve evaluation to guide targeted treatment, prevent deformities and improve mobility. Emerging information on neural correlates of neuromuscular impairments in CP provides the clinician with a more complete context with which to evaluate and develop effective treatment plans. Specifically, addressing the primary neuromuscular impairments and reducing secondary musculoskeletal impairments are important treatment goals. This perspective on neuromuscular mechanisms underlying gait abnormalities in spastic CP aims to inform clinical evaluation of CP, focus treatment more strategically, and guide research priorities to provide targeted treatments for CP.

## Introduction

Cerebral palsy (CP) is the most common movement disorder in children, with prevalence of 2–3/1,000 in general population and higher among preterm children (Oskoui et al., 2013). CP is diagnosed in young children based on abnormal posture or tone, delayed motor milestones, and gait abnormalities (Wu et al., 2004; Zhou et al., 2017). Typically gait stabilizes around age 4 years (Sutherland, 1997), highlighting importance of early identification and treatment. Spastic CP, the most common form of CP (Sellier et al., 2016), is characterized by four interrelated primary neuromuscular impairments: 1. muscle weakness, 2. short muscle-tendon units from slow muscle growth relative to skeletal growth, 3. muscle spasticity, and 4. impaired selective motor control (SMC). Identification neuromuscular impairments and their impact on gait can guide targeted treatment, as recent research indicates current treatment outcomes are modest (Schwartz et al., 2022) and more effective treatments are needed.

While 3D gait analysis is the gold standard for evaluating gait abnormalities in spastic CP (Gage and Novacheck, 2001), too often neuromuscular impairments of CP are not directly addressed. It is vital that clinical evaluation of CP systematically address impacts of primary neuromuscular and secondary musculoskeletal impairments on gait to target treatments. Here we discuss emerging information on neural correlates of neuromuscular impairments in spastic CP and their contribution to gait abnormalities to provide clinicians with more complete context to evaluate patients, develop effective treatment plans, and guide future research priorities.

## Brain injury in cerebral palsy

Cerebral palsy describes “a group of permanent disorders affecting the development of movement and posture, causing activity limitation, that are attributed to non-progressive disturbances that occurred in the developing fetal or infant brain” (Rosenbaum et al., 2007). While the initial brain injury is not progressive, the secondary musculoskeletal impairments and the impacts of the neuromuscular impairments on mobility are progressive (Bell et al., 2002; Sanger, 2015). The most common cause of CP is periventricular leukomalacia (PVL) with prematurity, intracerebral hemorrhage, and hydrocephalus (Metz et al., 2022). Other causes of brain injury include infection, metabolic disorders, brain malformation, and bilirubin neurotoxicity (Paneth et al., 2006; Strijbis et al., 2006; Ellenberg and Nelson, 2013; Zhou et al., 2017). Increased risk for CP is associated with multiple-births, such as twins and triplets (Sellier et al., 2021) and adverse neonatal outcomes are more common in males born preterm (Reid S. et al., 2016). Higher prevalence of CP with prematurity is in large part due to selective vulnerability to injury of developing white matter (neuron axons), during early phases of vascularization and white matter myelination (Inder and Volpe, 2000; Hoon et al., 2009). Approximately 30–80% of CP diagnoses with perinatal cause are secondary to genetic risk factors (Costeff, 2004; Moreno-De-Luca et al., 2012; MacLennan et al., 2015; Emrick and DiCarlo, 2020) and genetic testing is increasingly recommended (Pham et al., 2020; Emrick and DiCarlo, 2020; Gonzalez-Mantilla et al., 2023).

**FIGURE 1:**
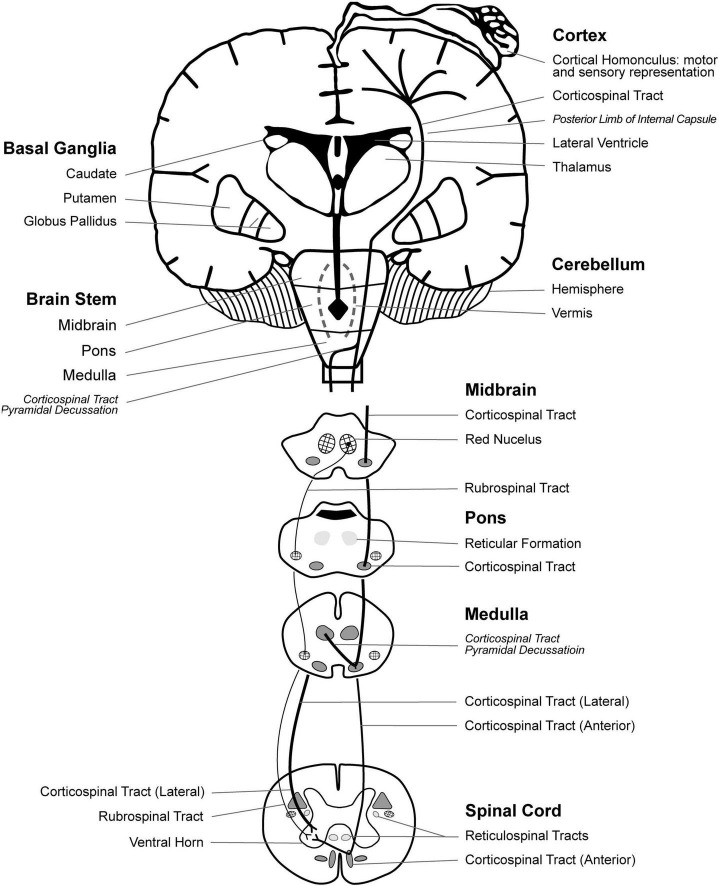
Neuroanatomical representation of brain motor regions, white matter tracts, and the motor and sensory homunculus. Revised with permission from Zhou et al. (2017).

Understanding the relevant neuroanatomy can assist with understanding pathophysiology of impairments resulting from the brain injury in CP. The cortical homunculus (Figure 1) illustrates the topographic representation of body regions in the cerebral cortex grey matter (neuron cell bodies) motor and sensory brain regions and descending white matter tracts (Nguyen and Duong, 2023). The corticospinal tract is the primary voluntary motor tract and originates in the cerebral cortex and descends in the pyramidal tract, named for its cross-sectional shape. In the medulla, about 75–90% of the fibers decussate, crossing over to create the lateral corticospinal tract, while the remaining fibers continue to descend making up the anterior corticospinal tract (Figure 1; Natali et al., 2018). The rubrospinal tract is one of several extra-pyramidal motor tracts that mediate involuntary movement; it originates in the red nucleus of the midbrain with decussation at the level of the midbrain (Figure 1; Lee and Muzio, 2020). The basal ganglia also mediate motor control and includes the caudate, putamen, and globus pallidus nuclei. The lateral ventricles are part of a system of ventricles that contain cerebral spinal fluid and provide nutrition, eliminate waste and protect the central nervous system (Scelsi et al., 2020).

Three types of CP, spastic, dyskinetic, and ataxic, arise from different regional brain injury. Spastic CP, the most common, affects approximately 87% of children with CP, while dyskinetic CP affects 7–15%, and ataxic CP affects 4% of children with CP (Sellier et al., 2016; Kitai et al., 2021). The different types of CP can co-exist but have characteristic motor deficits associated with specific regional brain injury.

Spastic CP is linked to damage to the corticospinal tract (CST) periventricular white matter due to prenatal hypoxia-ischemia (Liu et al., 2024). In addition, involvement of sensory-motor regions may further impair proprioception and motor function in spastic CP (Hoon et al., 2009). Spastic CP is further delineated by distribution of affected limbs: unilateral or bilateral.

Spastic CP is characterized by four primary interrelated neuromuscular impairments: 1. muscle weakness 2. short muscle-tendon units due to slow muscle growth relative to skeletal growth 3. muscle spasticity characterized by increased sensitivity to stretch, and 4. impaired selective motor control including flexor and extensor muscle synergies. Muscle weakness and short muscle-tendon unit result from reduced descending neural activation while muscle spasticity and impaired selective motor control (SMC) are thought to result from reduced descending inhibition.

Less severe involvement in spastic CP primarily affects distal joints, whereas more severe involvement affects distal and proximal joints, consistent with extent of brain injury and anatomical representation of cortical homunculus and descending motor tracts (Figure 1; Serdaroglu et al., 2004). Accordingly, Staudt et al. (2000) found correlations between lower limb motor function and extent of lateral lesion in the posterior semi-coronal plane on MRI. Recent evidence indicates motor impairments of spastic CP may be linked to both primary and downstream secondary abnormalities in the somatosensory cortex (Liu et al., 2024)

Neural correlates of gait abnormalities have been investigated in high-risk neonates and children with CP. Currently, structural MRI is standard for identifying neural injury in CP, however brain injuries are not always visible on structural MRI: a review by Reid et al. (2014) revealed 14% of children diagnosed with CP show no signs of brain injury on MRI, particularly in children with ataxic CP (Bax et al., 2006). Diffusion tensor imaging (DTI) provides more precise data on white matter tracts (Reid L. B. et al., 2016). Rose et al. (2007) found that near-term brain microstructure of posterior limb of internal capsule (PLIC) (Figure 1) on DTI in preterm neonates correlated with severity of gait abnormalities at 4 years, assessed with GMFCS and 3D gait kinematics. Rose et al. (2015) also reported that near-term cerebellar macrostructure on MRI, and PLIC and corpus callosum genu microstructure on DTI were predictive of neurodevelopment in toddlers assessed with Bayley Scales of infant Development and by gait temporospatial parameters. Meyns et al. (2016) found that severity of gait abnormality assessed using the Gait Profile Score correlated to total corpus callosum and subpart 1 volumes in children with CP. The Gait Profile Score is a measure based on kinematic parameters which compares the gait pattern of an individual to the mean pattern of normative typically developing group (Christian et al., 2017), similar to the Gait Deviation Index (Schwartz and Rozumalski, 2008). Fiori et al. (2014, 2015) developed a semiquantitative MRI score that correlated with severity of bilateral and unilateral CP as assessed with GMFCS. In a later study of unilateral CP, the semiquantitative MRI score correlated with gross manual dexterity as assessed with Manual Ability Classification Scale and Box and Block Score (Beani et al., 2024). These studies of brain structure-function relationships indicate a potential to identify young children at high risk for motor impairments that could guide early treatments.

Dyskinetic CP, the second most common type of CP, (Sellier et al., 2016; Kitai et al., 2021) is linked to damage of subcortical grey matter, including lower volumes of basal ganglia and thalamus, due to hypoxia-ischemia, as well as hyperbilirubinemia and birth asphyxia (Laporta-Hoyos et al., 2014; Himmelmann and Uvebrant, 2011; Kitai et al., 2021). The definition of dyskinetic CP has evolved to include dystonic and choreoathetoid CP (Aravamuthan and Waugh, 2016). As noted by Sanger (2006), symptoms of dyskinetic CP include both hyperkinetic and dystonic limb movements that impair function. Crichton et al. (2020) found basal ganglia and thalamus lesions on MRI were associated with reduced bimanual performance assessed with the Assisting Hand Assessment. Dyskinetic CP is often associated with GMFCS levels IV or V (Palisano et al., 2008; Himmelmann and Uvebrant, 2011). Milder cases may remain undiagnosed. Research on treatment for dyskinetic CP is needed. Early evidence indicates deep brain stimulation may be beneficial (Fehlings et al., 2024). Dyskinetic CP can co-occur along with spastic CP and delineation is important for appropriate treatment.

Ataxic CP is less common than spastic and dyskinetic CP and is linked with cerebellar vermis injury, cerebellar malformations, and/or genetic mutations (Zhou et al., 2017). Individuals with ataxic CP present with impaired coordination of voluntary movements and impairments to balance, stability, and speech (Hughes and Newton, 1992; Schnekenberg et al., 2015; Graham et al., 2016). The few studies of neurologic correlates of ataxic CP identified cerebral abnormalities and changes in the posterior fossa, involving the vermis of the cerebellum (Miller and Cala, 1989).

## Neuromuscular impairments of spastic cerebral palsy

Neuromuscular impairments differ among spastic, dyskinetic, and ataxic CP and are specific to the brain injury. Here we focus on neuromuscular impairments of spastic CP. In spastic CP, neurological injury to corticospinal tract results in four primary neuromuscular impairments: muscle weakness, short muscle-tendon unit, muscle spasticity, and impaired SMC. These primary neuromuscular impairments impact gait in specific ways and are important to delineate for targeted, effective treatment.

### Weakness

Muscle weakness in children with CP contributes to abnormal gait and posture (Brown et al., 1991; Damiano et al., 1995; Rose and McGill, 2005; Barber et al., 2012; Noble et al., 2014). Evidence indicates that decreased excitatory descending motor signals in the CST results in reduced neuromuscular activation and muscle volume that is exacerbated by secondary pathological changes in muscle elasticity (Rose and McGill, 2005; Stackhouse et al., 2005; Brown et al., 1991; Wiley and Damiano, 1998; Engsberg et al., 2000). Significant reduction in MVC of quadriceps, plantarflexors, and dorsiflexors occurs in children with CP (Damiano et al., 2002; Elder et al., 2003; Barber et al., 2012). Barber et al. (2012) found that children with CP have 33% lower ankle plantarflexion torque, explained in part by 33% smaller medial gastrocnemius. Rose and McGill (2005) found reduced MVC was associated with reduced neuromuscular activation and motor-unit recruitment in the medial gastrocnemius and tibialis anterior. Weakness in children with spastic CP is often under-recognized and contributes to gait abnormalities and functional limitations.

### Short muscle-tendon units

For children with spastic CP, short muscle results from slow muscle growth relative to skeletal growth, secondary muscle and fascia structural changes, and hypo- extensibility. Bi-articulate muscles, such as gastrocnemius, hamstrings, and rectus femoris, are most susceptible to joint contracture from rapid skeletal growth out-pacing muscle growth, which contributes to gait abnormalities. Barber et al. (2016) found reduced normalized muscle growth rate of medial gastrocnemius in CP. Similarly, Herskind et al. (2016) found lower muscle volumes in CP compared to age-matched TD children. Noble et al. (2014) also found children with CP had reduced volumes of lower extremity muscles compared to TD children, even when adjusted for body mass. In unilateral CP, Barrett and Lichtwark (2010) found affected side muscles were smaller than the unaffected side due to a lack of cross-sectional growth, not fascicle length. Thus, evidence indicates reduced muscle growth is a primary impairment in spastic CP.

While slow muscle growth relative to skeletal growth results in short muscle and is a disabling impairment in spastic CP, there is still limited understanding of the role of growth factors in CP. Smith et al. (2009) compared transcriptional profiles of muscle biopsy of six children with spastic CP and two TD children and noted competing upregulation of insulin-like growth factor 1 and myostatin, and aberrant regulation of excitation-contraction coupling genes. Loss of descending neural activation, reduced acetylcholine released at the neuromuscular junction, and altered action potentials within muscle all have potential to reduce muscle and nerve growth factors (Rose and McGill, 2005; Gough and Shortland, 2012), and warrant further investigations.

### Interrelation of weak and short muscle

Weak and short muscle are interrelated impairments in CP. Muscle force generation depends on quantity of cross-bridge formations and degree of myofilament overlap; maximal muscle force is produced at intermediate muscle lengths when myofilament overlap is optimal, but not at shortened or lengthened relative lengths (Gordon et al., 1966). Increased muscle sarcomere lengths in CP further contribute to muscle weakness due to inadequate myofilament overlap (Howard et al., 2022; Lieber et al., 2004)

Affected muscles in spastic CP have markedly reduced strength (Damiano et al., 2001); endurance, and neuromuscular activation (Rose and McGill, 2005; Eken et al., 2014) and an inability to recruit motor-units at higher firing rates (Rose and McGill, 2005). Weak, short muscles cause gait abnormalities that often progress as muscle growth fails to keep pace with skeletal growth. Baird et al. (2022) found 23% of participants with CP lost their ability to perform best walking by age 16, due to skeletal deformity, indicating slow muscle growth relative to skeletal growth contributes to gait deterioration.

Corticospinal tract input to the spinal cord begins prenatally and decreased input negatively impacts muscle innervation and activation as early as 17 to 29 weeks after conception (De Beukelaer et al., 2024). Thus, neonatal brain injury likely contributes to poor muscle growth and timing of brain injury during development may impact severity (Thorn et al., 2009; De Beukelaer et al., 2024). De Beukelaer et al. (2024) found children with spastic CP at 12mo of age had lower muscle volume and cross-sectional area of medial gastrocnemius than their TD peers, with lower growth rates found in GMFCS III and IV, compared to GMFCS II and I.

Dayanidhi and Lieber (2014) found 60–70% fewer satellite cells in muscle biopsied from children with CP compared to TD children, indicating satellite cells may play an important role in muscle growth and contracture. Increased stiffness of extracellular matrix and in vivo sarcomere length also contribute to muscle contracture (Smith et al., 2011; Lieber and Theologis, 2021). Increased passive muscle stiffness was proportional to thickened extracellular matrix-based collagen in children with CP (Howard et al., 2022), contributing to hypo-extensibility.

### Muscle spasticity

The neural-mediated reflex of muscle spasticity also creates resistance to muscle stretch and limits mobility. Bi-articulate and shortened muscles are more susceptible to influence of spasticity. Spasticity has been defined as “a velocity-dependent increase in muscle tone with exaggerated tendon jerks, resulting from hyper-excitability of the stretch reflex” (Mayer, 1997). Increased sensitivity to stretch in spastic CP is due to loss of inhibition to the muscle stretch reflex (Rose and McGill, 1998).

Despite muscle spasticity being a primary neuromuscular impairment of spastic CP, it is not well quantified (Bar-On et al., 2014a), often over-appreciated, and therefore its impact on gait is not well understood. A study by Choi et al. (2018) assessed hamstring strength and spasticity with the Modified Tardieu Scale and found that the R1 value of fast stretch, is more correlated to knee flexion in gait than R2 (PROM). Understanding how joint rotation impacts passive and active muscle response with precision can help delineate how spasticity impacts gait (Gao et al., 2009; Sloot et al., 2016; Lee et al., 2016). Bar-On et al. (2014b) quantified integrated biomechanical and EMG signals of manually performed passive stretches on medial hamstrings and gastrocnemius and found high measurement reliability, and significantly higher spasticity parameters in children with spastic CP than in TD children. Precise quantification of spasticity clinically and as a feature of musculoskeletal modeling can improve treatment specificity.

### Impaired selective motor control

Impaired SMC is defined as “impaired ability to isolate the activation of muscles in a selected pattern in response to demands of a voluntary posture or movement” (Sanger, 2006) including muscle synergies and mirror movements (Rose, 2009; Cahill-Rowley and Rose, 2014; Fahr et al., 2020). Impaired SMC has been assessed using the SCALE, an observation-based measure for children with spastic CP which correlates with GMFCS (Fowler et al., 2009). Impaired SMC assessed with SCALE correlates with knee flexion at IC, short step length, and decreased velocity (Rha et al., 2016). Children with spastic CP demonstrate obligatory co-activation of quadriceps and gastrocnemius on EMG, which distinguishes mild spastic CP from idiopathic toe walking (Rose et al., 1999; Policy et al., 2001). Reduced complexity of neuromuscular control during gait on EMG was found in CP (Steele et al., 2015). Yun et al. (2023) evaluated 36 children with bilateral spastic CP, GMFCS I-II, and found associations between impaired SMC on SCALE and lower scores on GMFM-88, Pediatric Balance Scale, Edinburg Visual Gait Scale,10-Meter Walk Test, and Timed Up and Go Test. Additionally, Noble et al. (2019) studied 11 children with CP and found strong correlations between GMFM- 66 with SCALE, muscle volume on MRI, and spasticity assessed on Modified Ashworth Scale. Recently, Graci et al. (2024) developed indices for coactivation, mirror movement, synergy, and overflow for children with CP using EMG for quantify and classify impaired SMC to guide targeted interventions.

Spared “extrapyramidal” motor tracts, such as the rubrospinal and reticulospinal tracts, may provide imperfect compensation in recovering motor function after corticospinal tract injury (Yeo and Jang, 2010; Rüber et al., 2012). As shown in Figure 1, the corticospinal tract originates in the motor cortex, whereas the rubrospinal tract originates in the mid brain (red nucleus), descends along with the corticospinal tract, and mediates flexion and extension movements (Mtui et al., 2016). Evidence also suggests that there is overlap of the terminating fibers of the corticospinal tract and rubrospinal tracts in the spinal cord grey matter of humans (Olivares-Moreno et al., 2021). The rubrospinal tract is better developed in infants than in children and adults. However, in stroke the rubrospinal tract re-develops, as assessed on DTI, associated with impaired SMC movement patterns post-corticospinal tract injury (Yeo and Jang, 2010; Rüber et al., 2012). Cortical mapping with electroencephalographic and torque metrics in stroke patients suggests that impaired SMC is associated with increased overlap of joint representation in sensorimotor cortices (Yao et al., 2009). Further research can characterize development and function of the rubrospinal and reticulospinal tracts in spastic CP and potential for plasticity to improve SMC.

## Neuromuscular impairments and gait in spastic cerebral palsy

**FIGURE 2:**
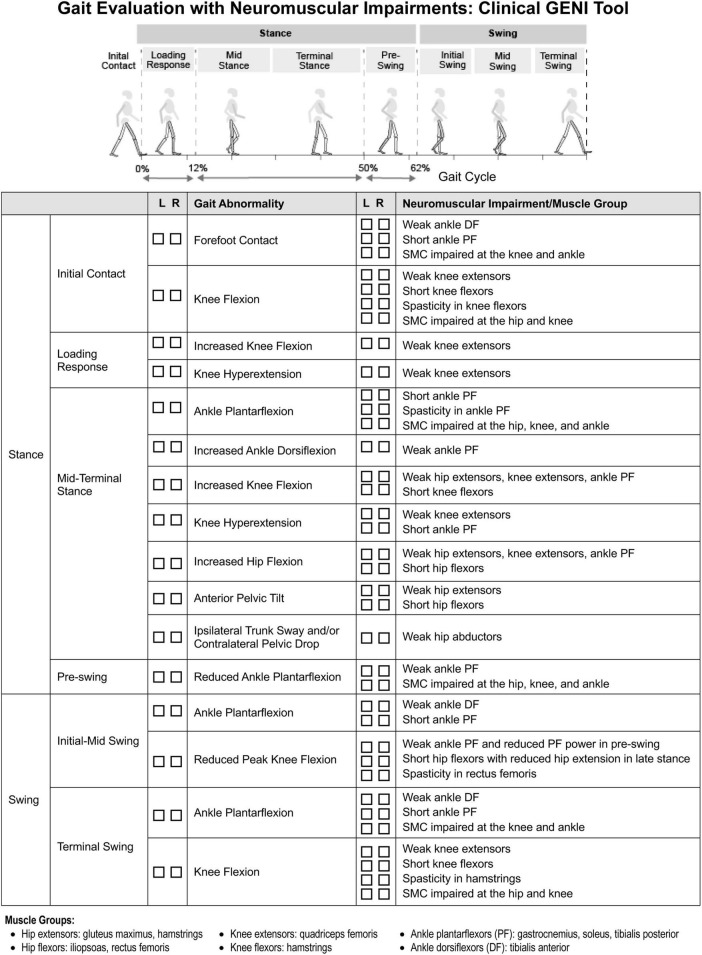
Clinical gait evaluation with consideration of neuromuscular impairments. Revised with permission from Zhou et al. (2017).

Figure 2 shows a gait assessment with the neuromuscular impairments that contribute to specific gait events in spastic CP. This delineation can guide evidence-based, targeted treatment to better improve gait. Figure 2 can also be used clinically as a checklist to help identify the contributions of neuromuscular impairments to gait.

### Weakness: gait abnormalities and treatment considerations

Muscle weakness impacts both stance and swing phases of gait (Figure 2). At initial contact (IC), weak ankle dorsiflexors (DF) contribute to ankle plantarflexion and foot-slap. In stance, weak ankle plantarflexors (PF), knee extensors and hip extensors contribute to flexed hip, knee, and ankle gait pattern. When the foot is planted on the ground, both the hip and ankle extensors act to extend the knee, through their actions on the femur and tibia respectively, along with the knee extensors. Quadriceps weakness can also contribute to knee hyperextension in stance, compensatory to reduce demand on quadriceps. In pre-swing as the ankle transitions from DF to PF, weak ankle PF decreases ankle PF power which reduces peak knee flexion in swing. Weak ankle DF contributes to ankle PF and poor foot clearance in swing. In terminal swing, weak knee extensors limit knee extension, causing knee flexion in terminal swing and IC. Steele et al. (2012) found children with flexed gait had less passive skeletal support of body weight and utilized higher active muscle forces to walk than TD children.

Proximal stability is critical for independent gait: weak trunk and pelvic musculature can contribute to ipsilateral trunk sway and contralateral pelvic drop in stance, trunk and pelvic rotation, and increased anterior pelvic tilt with lumbar lordosis.

Interventions targeting weak muscles are effective at improving strength and mobility without adverse effects in CP (Dodd et al., 2002; Scholtes et al., 2012). While evidence is accumulating on ideal dosing of strength training programs, additional research is needed. Cho and Lee (2020) conducted a randomized control trial with 25 children with spastic CP performing functional progressive resistance exercise for 30 minutes, 3x/wk, for 6 weeks and found improved strength assessed with hand-held dynamometer, increased rectus femoris cross-sectional area and quadriceps thickness on ultrasound imaging, and improved functional ability on GMFM-88. Verschuren et al. (2016) reviewed dosing recommendations for exercise and physical activity for CP. A scoping review by Mooney and Rose (2019) found weakness and spasticity were improved with single-channel neuromuscular electrical stimulation (NMES) during gait for children with CP. More research is needed on strengthening and multi-channel NMES to develop effective treatment that translate to improved gait.

### Short muscle and hypo-extensibility: gait abnormalities and treatment considerations

Short ankle PF contribute to forefoot IC and ankle PF in stance and swing (Figure 2). Short tibialis posterior contributes to plantarflexed-varus posture. Over time, short PF increase midfoot breakdown, reduce pre-swing PF power, and reduce foot-floor clearance in swing. Rha et al. (2016) reported short medial gastrocnemius correlated with knee flexion angle at IC. Additionally, short hamstrings limit terminal swing knee extension causing knee flexion at IC. Arnold et al. (2006) reported two-thirds of flexed-knee gait cases were related to short semitendinosus at IC and single leg stance based on musculoskeletal modeling. Short rectus femoris can limit knee flexion in swing. Normally, passive structures store elastic energy and act as a spring, such as hip flexors in pre-swing, as highlighted in a systematic review by Koussou et al. (2021). Consequently, short hip flexors limit hip extension in terminal stance, dampen the spring, and reduce knee flexion in swing. Additionally, short adductors contribute to increased hip adduction, narrow step width, and scissoring gait.

The challenge is, how to address short muscle while also preserving strength? Given the pennate fiber angle of gastrocnemius, strengthening which increases muscle fiber diameter, can increase muscle length across joint axes (Shortland et al., 2001; Gough and Shortland, 2012). Kalkman et al. (2019) conducted a randomized controlled trial which found resistance training with passive stretching improved tendon stiffness and muscle fascicle length in CP. Additionally, eccentric strengthening improves ROM and strength (O’Sullivan et al., 2012).

Research indicates that soft-tissue surgery, such as tendo-Achilles lengthening and gastrocnemius recession, may reduce muscle strength (Wren et al., 2010). This may in part may explain modest outcomes of surgery for children with spastic CP (Schwartz et al., 2022). Howard et al. (2022) reported with gastrocnemius-soleus recession, there was decreased fascicle length, increased pennation angle with no change in muscle volume; however, a reduction in muscle volume was found after surgery to semitendinosus. Additionally, serial casting contributes to muscle weakness due to prolonged immobilization. Further research is needed to improve clinical monitoring of muscle growth relative to skeletal growth, such as with ultrasound, to better target and evaluate interventions. In the absence of ultrasound, for example, using a tape measure for upper and lower leg length measurement in relation to muscle girth at a percent distance from boney landmark may offer valuable clinical monitoring of muscle growth.

### Spasticity: gait abnormalities and treatment considerations

Spasticity most prominently impacts bi-articulate muscles, which experience greater stretch excursion. Short muscles, which experience stretch more frequently. However, the effect of spasticity on gait is often overestimated, whereas short muscle and structural hypo-extensibility may be under appreciated. Similar to short muscle, spasticity of gastrocnemius can contribute to forefoot IC and ankle PF in stance, with rapid stretch during the ankle rocker (Figure 2). Hamstring spasticity can contribute to limited knee extension in terminal swing and knee flexion at IC and stance (Steele et al., 2012). Additionally, spasticity of rectus femoris can contribute to reduced knee flexion in early swing, decreased peak knee flexion, limiting foot-floor clearance. Hip adductor spasticity can contribute to adducted, scissoring gait pattern.

BoNT-A injections, baclofen, and selective dorsal rhizotomy are common treatments for spasticity, but have risks, including muscle weakness (Walhain et al., 2021). There is some evidence that non-pharmacologic and non-surgical interventions can be effective at reducing spasticity while also preserving strength. Williams et al. (2013) found BoNT-A combined with strength training reduced spasticity, and improved strength when compared to BoNT-A alone in 15 children with spastic diplegic CP aged 5–12 years. A study conducted by Mahmood et al. (2024) found spasticity was reduced after six and 12 weeks of high dosed massage home program. Further research is needed to assess the efficacy of treatments that promote muscle strength and length, reduce spasticity, and improve gait.

### Impaired selective motor control: gait abnormalities and treatment considerations

Impaired SMC arises from in obligatory synergistic co-activation and impacts the gait cycle when the hip, knee, and ankle are required to flex or extend independently. For example, impaired SMC contributes to ankle PF in terminal swing and IC as the knee extends and also contributes to ankle PF in stance as the hip and knee extend (Figure 2). Impaired SMC can also reduce pre-swing push-off mechanics by reducing ankle PF as the hip and knee begin to flex, slowing the transition into hip and knee flexion in swing. Additionally, impaired SMC can contribute to knee flexion in terminal swing due to hip flexion. A study conducted by Rha et al. (2016) found that impaired SMC, assessed with SCALE, was a stronger correlate of knee flexion at IC than estimated length of semitendinosus assessed with musculoskeletal modeling.

Research investigating impaired SMC is limited, however exercises promoting movement patterns outside of synergy may prove beneficial and warrant further study. A systematic review by Fahr et al. (2020) found existing interventions had variable efficacy in improving SMC. There is indication that younger children with CP may have greater sensitivity to interventions addressing muscle synergies due to higher brain plasticity (Yang et al., 2013; Bekius et al., 2020). Research is needed to determine if treatment can improve SMC and translate to improved gait.

Of note, Figure 2 addresses gait abnormalities that occur during specific phases of gait and are directly related to neuromuscular impairments of spastic CP. Figure 2 does not address gait abnormalities directly related to balance impairment, such as wide-base gait, or those directly related to pain, such as short stance phase of antalgic gait and ipsilateral trunk sway, or those directly related to skeletal deformities and leg length discrepancies which also can contribute to gait abnormalities, including abnormal foot progression angle, in spastic CP. It also does not address gait abnormalities that are compensatory, such as increased hip and knee flexion of steppage gait which compensates for ankle plantarflexion in swing. These impairments and compensations need to be delineated in order to guide effective treatment.

## Conclusion

This perspective reviewed links between brain injury, neuromuscular impairments and resulting gait abnormalities in spastic CP. By providing this context, we hope to encourage recognition of the neuromuscular and musculoskeletal mechanisms underlying gait abnormalities in CP and focus research to better identify and quantify these impairments, delineate their contribution to gait abnormalities, and develop targeted treatments. For example, research as well as regular clinical assessment of muscle growth relative to skeletal growth would better quantify this consequential impairment and help develop standardized outcome measures. Additionally, future research should focus on how muscle growth, especially in bi-articulate muscles, affects the impact of spasticity on gait in order to better focus treatments. Expanding the features of musculoskeletal modeling and artificial intelligence applications to include neuromuscular impairments, such as impaired SMC. and discover targeted treatment opportunities that can address muscle growth and SMC to substantially improve functional outcomes for individuals with CP.

## Data Availability

The original contributions presented in the study are included in the article/supplementary material, further inquiries can be directed to the corresponding author.
